# Supplementary material for: Enantiomer-selective magnetization of conglomerates for quantitative chiral separation
Source: Nat Commun. 2019 Apr 29;10:1964. doi: 10.1038/s41467-019-09997-y (PMC6488659; doi:10.1038/s41467-019-09997-y)
Supplement: Supplementary file 1 — Supplementary Information [file 41467_2019_9997_MOESM1_ESM.pdf]

Enantiomer-selective magnetization of conglomerates for quantitative chiral separation

Xichong Ye,[1†] Jiaxi Cui,[2,3†] Bowen Li,[1] Na Li,[1] Rong Wang,[1] Zijia Yan,[1] Junyan Tan,[1] Jie Zhang,[1] Xinhua Wan[1*]

[1] Beijing National Laboratory for Molecular Sciences, Key Laboratory of Polymer Chemistry and Physics of Ministry of Education, College of Chemistry and Molecular Engineering, Peking University, Beijing 100871, China.[2] INM - Leibniz Institute for New Materials, Campus D2 2, 66123, Saarbrucken, Germany.[3] Institute of Fundamental and Frontier Sciences, University of Electronic Science and Technology of China, Chengdu 611731, China.

[†] These authors contributed equally to this work.

*Corresponding author: xhwan@pku.edu.cn (X. H. Wan)

Supplementary Information

■ Supplementary Notes

Supplementary Note 1. Materials: Methacryloyl chloride (97%, Energy Chemical), *N*-α-(*tert*-butoxycarbonyl)-*S(R)*-lysine (98%, Energy Chemical), boc-4-amino-*S*-phenylalanine (98%, Energy Chemical), *N*-(3-dimethylaminopropyl)-*N'*-ethylcarbodiimide hydrochloride (EDC, 98%, Heowns), trimethylsilyldiazomethane (in hexane, 2 M, Alfa-Aesar), 1,2-bis(2-aminoethoxy)ethane (98%, J&K Co.), dibenzyl ether (95%, Macklin), iron (III) acetylacetonate (98%, J&K Co.), 1,2-hexadecanediol (98%, J&K Co.), oleylamine (90%, Energy Chemical), oleic acid (90%, TCI), toluene-4-sulfonic acid monohydrate (*p*Ts•H_2O, 98%, Sinopharm Chemical Reagent Co.), (2*S*,3*R*)-threonine (*S*-Thr), (2*R*, 3*S*)-threonine (*R*-Thr), *R*-asparagine monohydrate (*R*-Asn•H_2O), and *S*-asparagine monohydrate (*S*-Asn•H_2O) (98%, Alfa Aesar), (2*S*, 3*S*)-allo-threonine (*S*-aThr), (2*R*, 3*R*)-allo-threonine (*R*-aThr) (99%, HarveyBio), *R*-4-hydroxyphenylglycine (*R*-*p*Hpg) and *S*-4-hydroxyphenylglycine (*S*-*p*Hpg) (98%, J&K Co.) were used as purchased. Styrene (St, AR, 10-15 ppm TBC as stablilizer, Macklin) was purified through Al_2O_3 column chromatography, dried with calcium hydride and distilled out under vacuum at 35 ^{o}C. Azobisisobutyronitrile (AIBN, AR, Wuhan Chemical Co.) was recrystallized

three times from ethanol and dried under vacuum at room temperature. Dioxane and tetrahydrofuran (AR, Beijing Chemical Co.) were refluxed with sodium and distilled before use. Dichloromethane were distilled out from P_2O_5. 4-Cyano-4-[(dodecylsulfanylthiocarbonyl)sulfanyl]pentanoic acid (CDP, 98%, Sigma-Aldrich) was recrystallized from hexane, and stored at 4 ^oC.

Supplementary Note 2. Measurements: Fourier transform infrared (FTIR) spectra were recorded on a PE 100 spectrometer with a disc of KBr. ^{1}H NMR experiments were carried out on a Bruker ARX400 spectrometer at room temperature using TMS as an internal standard. Dynamic light scatter (DLS) measurements were performed on a commercialized spectrometer from Brookhaven Instrument Corporation (BI-200SM Goniometer, Holtsville, NY). A vertically polarized, 100 mW solid-state laser (GXC-III, CNI, Changchun, China) operating at 633 nm was used as the light source, and a BITurboCo digital correlator (Brookhaven Instruments Corp.) was used to collect and process data. The samples were filtered through 450 nm filters. TEM images were obtained on a JEM-2010 (JEOL, Japan) transmission electron microscopy operated at 200 KV. The samples were prepared by dipping a drop of solution onto copper grids coated with amorphous carbon membranes and then drying in air. The magnetic properties of the magnetomicelles were measured by PPMS-9 SQUID magnetometer (Quantum Design) with fields up to 2 Tesla. The solid state magnetomicelles were placed in capsules. The magnetic moment as a function of applied magnetic field was measured at 298 K. The number-average molecular weights (M_n), weight-average molecular weights (M_w), and polydispersity indices (PDI=M_w/M_n) of the resultant polymers were estimated on a gel permeation chromatographic (GPC) instrument equipped with a Waters 515 HPLC pump and a Waters 2410 refractive-index detector. Three Waters Styragel columns with 10 mm bead size were connected in tandem. Their effective molecular weight ranges were 100-10000 for Styragel HT2, 500-30000 for Styragel HT3, and 5000-600000 for Styragel HT4, respectively. The pore sizes were 50, 100, and 1000 nm for Styragels HT2, HT3, and HT4, respectively. THF was used as the eluent at a flow rate of 1.0 mL min^{-1} at 35 ^oC. The calibration curve was obtained against polystyrene standards. Thermogravimetric Analysis (TGA) were recorded on Q600 TGA-DSC-DTA (TA Instruments). Dry powders were placed in ceramic crucible and analyzed over the temperature range of room temperature to 600 °C at the rate of 10 ^oC min^{-1} under dry flow of N_2 at a rate of 100 mL min^{-1}.

A J-810 circular dichroism spectrometer (Jasco Corporation, Japan) was involved to achieve the circular dichroism spectra.

The ee% was also estimated on a high performance liquid chromatography (HPLC) equipped with a JASCO PU-2089 pump, a AS-2055 automatic sampler, a UV-2070 UV-Vis spectrometer, a CD-2095 circular dichroism spectrometer and a Daicel CROWNPAK CR(+) column. Perchloric acid aqueous solution was used as the eluent (Asn: pH = 1.5; flow rate, 0.4 mL min^{-1}; temperature, 7 ^{o}C. Thr: pH = 2.0; flow rate, 0.4 mL min^{-1}; temperature, 7 ^{o}C. aThr: pH = 1; flow rate, 0.4 mL min^{-1}; temperature, 7 ^{o}C. *p*Hpg*p*Ts, pH = 1; flow rate, 0.6 mL min^{-1}; temperature, 7 ^{o}C).

■ Supplementary Methods:

Synthesis of *N*^{2}-(*tert*-butoxycarbonyl)-*N*^{6}-methacryloyl-*S*-lysine (*S*-MALBoc). *α*-(*tert*-Butoxycarbonyl)-*S*-lysine (10.0 g, 40.6 mmol) was dissolved in 70 mL of water. The solution of the methacryloyl chloride (5.1 g, 48.8 mmol) in 48 mL of dry THF was added dropwise into the *α*-(*tert*-butoxycarbonyl)-*S*-lysine solution at 0 ^{o}C with vigorous stirring, and keeping the pH value close to 9 by gradual addition of 1 M NaOH aqueous solution. Keep stirring at room temperature for 1 day, followed by washing with 3 × 150 mL portions of diethyl ether. The aqueous solution was acidified with 0.5 M HCl until pH close to 3. Then the mixture was extracted with 3 × 150 mL portions of ethyl acetate. The organic layers were combined, and dried over anhydrous Na_2SO_4. After evaporation of the solvent under reduced pressure, the crude product was purified further by column chromatography (silica gel, dichloromethane/methanol (20/1, v/v) as eluent) and recrystallized in CH_2Cl_2 to give 10.2 g of white solids. Yield: 80 %. *R*-MALBoc was prepared by a similar way.

^{1}H NMR (400 MHz, $CDCl_3$, δ, ppm): 1.44 (m, 11H; -C*(CH_3)$_3$* & -CH_2CH_2*CH_2*CH_2CH-), 1.55-1.62 (m, 2H; -CH_2*CH_2*CH_2CH_2CH-), 1.70-1.87 (m, 2H; -CHCH_2CH_2*CH_2*CH-), 1.95 (s, 1H; =C(*CH_3*)-), 3.29-3.34 (m, 2H; -*CH*$CH_2CH_2CH_2$CH-), 4.12-4.29 (m, 1H; -CH$CH_2CH_2CH_2$*CH*-), 5.30-5.35 (m, 2H; vinyl & NH), 5.70 (s, 1H; vinyl), 6.13-6.23 (m, 1H; NH), 9.94 (broad, 1H; -COOH).

Synthesis of *p*-methacrylamido-*α*-*tert*-butoxycarbonyl-*S*-phenylalanine. ((*S*)-MPABoc). Boc-4-amino-*L*-phenylalanine (5.0 g, 17.8 mmol) was dissolved in 65 mL of water. The solution of the methacryloyl chloride (2.2 g, 21.1 mmol) in 40 mL of dry THF was added dropwise into the solution at 0 ^{o}C with vigorous stirring, and keeping the pH value close to 9 by gradual addition of 1 M NaOH aqueous solution. Keep stirring at room temperature for 1 day, followed by washing with 3 × 150 mL portions of diethyl ether. The aqueous solution was acidified with 0.5 M HCl until pH close to 3. Then the mixture was extracted with 3 × 150 mL portions of ethyl acetate. The organic layers were combined, and dried over anhydrous Na_2SO_4. After evaporation of the solvent under reduced pressure, the crude product was purified further by column chromatography (silica gel, dichloromethane/methanol (20/1, v/v) as eluent) and recrystallized in CH_2Cl_2 to give 4.9 g of white solids. Yield: 78 %.

^1H NMR (400 MHz, DMSO-d_6, δ, ppm): 1.33 (s, 9H; -C*(CH₃)₃*), 1.94 (s, 1H; =C(*CH₃*)-), 2.78-2.95 (m, 2H; Ar-*CH₂*-), 4.04 (m, 1H; -CH₂-*CH*(COOH)-), 5.49-5.77 (d, 2H; vinyl), 7.07 (s, 1H; NH), 7.18-7.56 (m, 4H; Ar-H), 9.71 (s, 1H; NH), 12.58 (s, 1H; -COOH).

Synthesis of CDP-terminated Poly[N^2-(*tert*-butoxycarbonyl)-N^6-methacryloyl-*S*-lysine] (CDP-*S*-PMAlBoc). A typical process for the polymerization was presented as follows: MALBoc (0.95 g, 3.0 mmol), AIBN (0.98mg, 0.0060 mmol), CDP (24.2mg, 0.060 mmol), and dioxane (6.0 g) (molar ratio of MALBoc/CDP/AIBN = 500/10/1) were introduced into a polymerization tube with a magnetic bar. After three freeze-pump-thaw cycles, the tube was flame-sealed under vacuum and put into an oil-bath thermostatted at 65 ^{o}C. After the polymerization continued for 10 h, the tube was cooled to room temperature and broken. The solution was diluted with 10 mL of THF and added dropwise into 150 mL of diethyl ether. The precipitated solids were collected by filtration and dried under vacuum for 24 h at room temperature to give 0.45 g of yellow powders. Yield: 48%.

^1H NMR (400 MHz, DMSO-d_6, *δ*, ppm): 0.7-1.6 (-C(*CH₃*), $-CH_3$, main chain CH_2, and CH_2 in Lys), 2.9-3.5 (CH_2 in Lys), 3.7-3.9 (CH in Lys), 6.6-7.2 (NH), and 12.4 (COOH). FTIR (neat, KBr plate, wavenumber, cm^{-1}): 3393, 2978, 2936, 2869, 1711, 1646, 1527, 1457, 1394, 1367, 1251, 1168.

Synthesis of CDP-terminated Poly[*p*-methacrylamido *tert*-butoxycarbonyl-*S*-phenylalanine] (CDP-*S*-PMPABoc). A typical process for the polymerization was presented as follows: MPABoc (3.5 g, 10.0 mmol), AIBN (3.3 mg, 0.020 mmol), CDP (40.4 mg, 0.10 mmol), and dioxane (35.0 g) (molar ratio of MPABoc/CDP/AIBN = 500/5/1) were introduced into a polymerization tube with a magnetic bar. After three freeze-pump-thaw cycles, the tube was flame-sealed under vacuum and put into an oil-bath thermostatted at 80 ^{o}C. After the polymerization continued for 48 h, the tube was cooled to room temperature and broken. The solution was diluted with 30 mL of THF and added dropwise into 800 mL of diethyl ether. The precipitated solids were collected by filtration and dried under vacuum for 24 h at room temperature to give 2.84 g of yellow powders. Yield: 81%. After methyl esterification, the polymer is tested by GPC, the M_n is 15703, and the PDI is 1.28.

^{1}H NMR (400 MHz, DMSO-d_6, δ, ppm): 0.4-1.5 (-C(CH_3), -CH_3, main chain CH_2), 2.5-3.3 (CH_2 and CH in phenylalanine), 6.4-6.8 (NH), 6.8-7.7 (Ar-H), 8.6-9.4 (NH) and 12.4-12.7 (COOH).

RAFT polymerization of St in the presence of CDP-*S*-PMALBoc. CDP-*S*-PMALBoc, St, AIBN, and methanol with the molar ratio of St/PMALBoc-CDP/AIBN 10000/10/1 and the weight ratio of St/dioxane 0.30 g/1.00 g were added into a polymerization tube containing a magnetic bar. The mixture was degassed by three freeze-pump-thaw cycles. The tube was flame-sealed under vacuum and put into an oil-bath thermostatted at 80 ^{o}C with stirring. The polymerization degree of PSt can be adjusted by controlling the reaction time. After polymerization was carried out for the prescribed time, the tube was placed into ice bath to stop the reaction. The solution was diluted with THF and added dropwise into diethyl ether. The precipitated solids were collected by filtration and dried under vacuum for 24 h at room temperature to give white powders.

^{1}H NMR (400 MHz, DMSO-d_6, δ, ppm): 0.7-2.1 (main chain CH_2, and CH_2 in Lys), 2.7-3.5 (main chain CH, and CH_2 in Lys), 3.6-4.0 (CH in Lys), 5.8-9.0 (Ar-H and NH).

RAFT polymerization of St in the presence of CDP-*S*-PMPABoc. CDP-*S*-PMPABoc, St, AIBN, and methanol with the molar ratio of St/PMALBoc-CDP/AIBN 10000/10/1 and the weight ratio of St/dioxane 0.16 g/1.00 g were added into a polymerization tube containing a magnetic bar. The mixture was degassed by three freeze-pump-thaw cycles. The tube was flame-sealed under vacuum and put into an oil-bath thermostatted at 80 ^oC with stirring. After polymerization was carried out for the prescribed time, the tube was placed into ice bath to stop the reaction. The solution was diluted with THF and added dropwise into diethyl ether. The precipitated solids were collected by filtration and dried under vacuum for 24 h at room temperature to give white powders.

^1H NMR (400 MHz, DMSO-d_6, δ, ppm): 0.4-1.5 (-C(CH_3), -CH_3, main chain CH_2), 2.6-3.3 (CH_2 in phenylalanine), 3.8-4.2 (CH in phenylalanine), 6.3-6.8 (NH), 6.8-7.7 (Ar-H), 8.6-9.3 (NH) and 12.4-12.8 (COOH).

Deprotection of N-Boc group (PSt-*b*-*S/R*-PMALHCl and PSt-*b*-*S/R*-PMPAHCl). Polymer (500 mg) and THF (30 mL) was added into a 50 mL flask, and keep stirring for 1 h until the solution become homogeneous. Hydrochloric acid (10 M, 3 mL) was added dropwise into this solution, and then the mixture was stirred for 6 h at room temperature, and a gelatinous sediment appeared. After pouring the solvent out, 50 mL diethyl ether was added to the crude product and stirred for 1 h. Pouring the solvent out and repeat this process for 5 times. The precipitated solids were filtered and then dried under vacuum at 35 ^oC for 12 h. Yield: ~100%.

Self-assembly of diblock copolymers and crosslinking of shell. Freshly prepared 4 mL PSt-*b*-PMAL stock solution (10 mg mL^{-1} in DMSO) was diluted with 36 mL of DMSO and 40 mL THF slowly with vigorous stirring, such that $[copolymer]_{initial}$ = 0.50 mg mL^{-1} in 50:50 DMF/THF. Then, 320 mL of H_2O was gradually added dropwise to the solution at a rate of 0.5 mL min^{-1} with vigorous stirring. As water was added, the solution gradually turned a light milky suspension in color, indicating the formation of micelle suspension. The PMAL blocks of the resulting micelles were cross-linked by addition of 2,2'-(ethylenedioxy)bis(ethylamine) as a difunctional linker and EDC as an activator. For 30% crosslinking of the PMAL blocks in a typical dispersion, the carboxylic acid groups on the PMAL/PMPA blocks were first activated by

adding 1.44 mL of freshly prepared EDC solution (1.0 wt% in DMF). The resulting suspension was left to react with stirring for 30 min. The cross-linking of the outer shells of micelles was completed by adding 0.7 mL 2,2'-(ethylenedioxy)bis(ethylamine) solution (1.0 wt% in DMF) in one portion with stirring. After stirring for 2 h, the resulting suspension was subjected to the dialysis against DI water for over 72 h (Genia Biotech® Regenerated Cellulose Membrane, MWCO = 3500 Da) to remove any residual solvent and unreacted small molecules. 2.0 µL of the solution was deposited on the TEM grid, dried in air before taking TEM images.

Calculation of loaded MNPs. The number of loaded MNPs in each micelle can be estimated through the equation E1[1], where D_{MNPs} is the mean diameter of Fe_3O_4 MNPs, D_{micelle} is the mean diameter of the hybrid micelle, and φ_{MNP} is the volume fraction of Fe_3O_4 MNPs in micelles. The mean value of the D_{MNPs} and D_{micelle} were obtained from TEM measurement. The values of φ_{MNP} were calculated from the equation E2, where M is the weight and ρ is the density of each part in the micelle. The ρ_{organic} can be calculated from the equation E3. The weight percentage of each part can be obtained from TGA results. The ρ_{PSt}=1.06 g cm^{-3}; ρ_{PMAL}=1.37 g cm^{-3}; $\rho_{\mathrm{Oleic\ acid}}$=0.89 g cm^{-3}; ρ_{MNPs}=5.18 g cm^{-3}. The calculation results were summarized in table S3.

$$N=\frac{4}{3}\pi\left(\frac{D_{micelle}}{2}\right)^3\varphi_{MNP}/\frac{4}{3}\pi\left(\frac{D_{MNP}}{2}\right)^3 \qquad (1)$$

$$\varphi_{MNP}=\frac{\sum V_{MNP}}{\sum V_{total}}\times 100\%=\frac{\sum M_{MNP}/\rho_{MNP}}{\sum M_{MNP}/\rho_{MNP}+\sum M_{organic}/\rho_{organic}}\times 100\% \qquad (2)$$

$$\rho_{organic}=\frac{m_{tol}}{V_{tol}}=\frac{m_{tol}}{\frac{m_{PSt}}{\rho_{PSt}}+\frac{m_{PMAL}}{\rho_{PMAL}}+\frac{m_{Oleic\ acid}}{\rho_{Oleic\ acid}}}=\frac{1}{\frac{\omega_{PSt}}{\rho_{PSt}}+\frac{\omega_{PMAL}}{\rho_{PMAL}}+\frac{\omega_{Oleic\ acid}}{\rho_{Oleic\ acid}}} \qquad (3)$$

■ Supplementary Figures

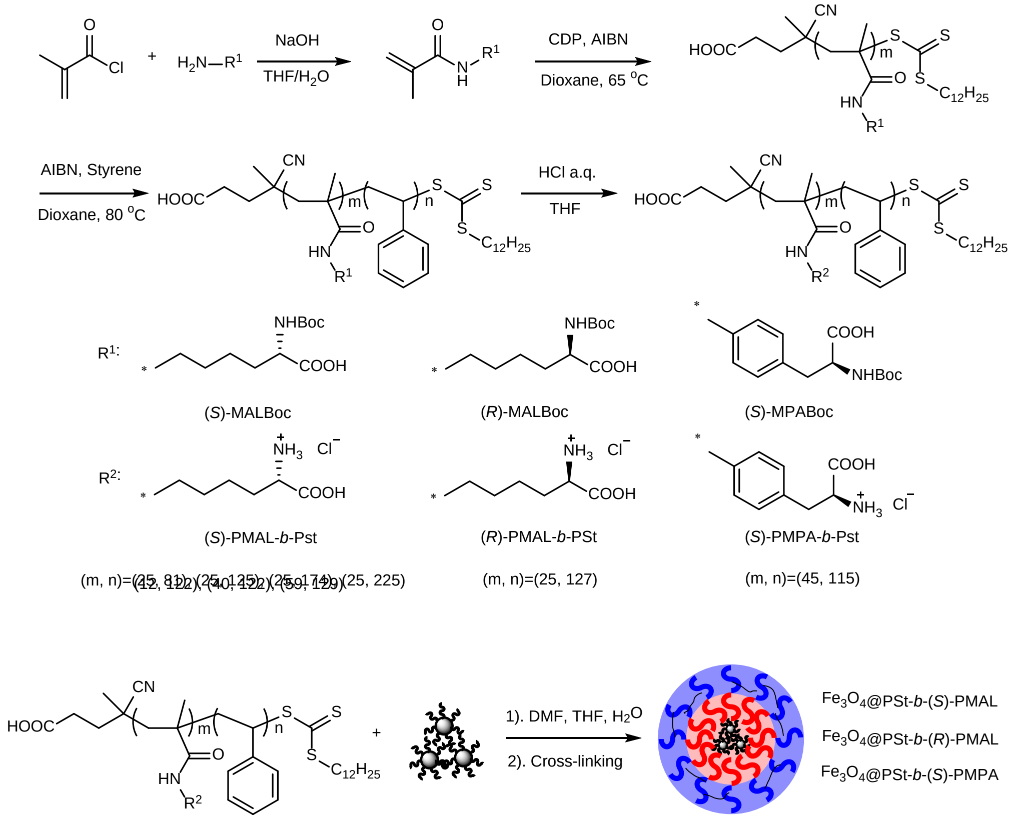

Supplementary Figure 1. Synthesis strategy of nano-splitters.

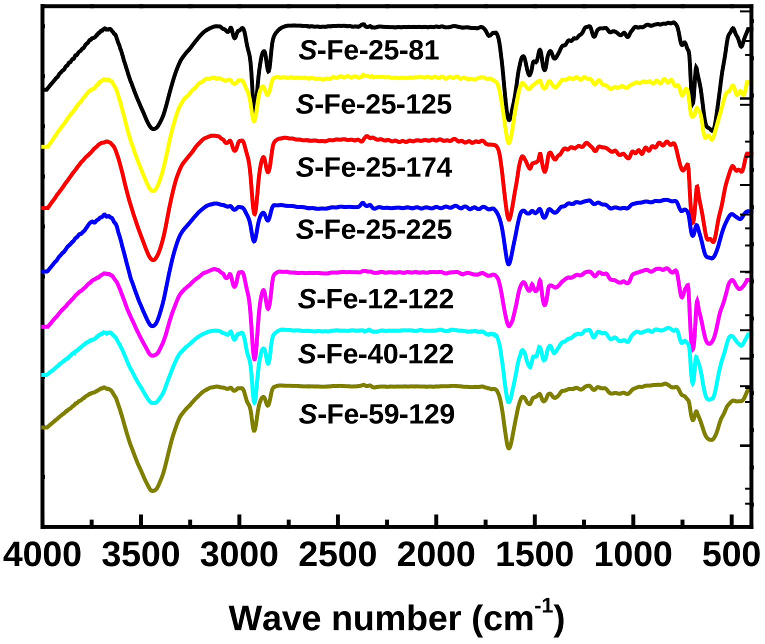

Supplementary Figure 2. FTIR spectra of *S*-Fe-m-n

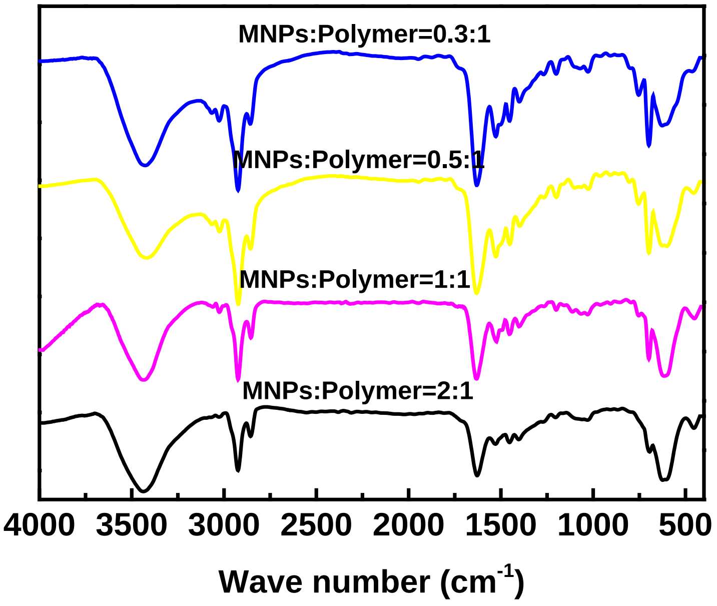

Supplementary Figure 3. FTIR spectra of *S*-Fe-40-122 with different composite ratio.

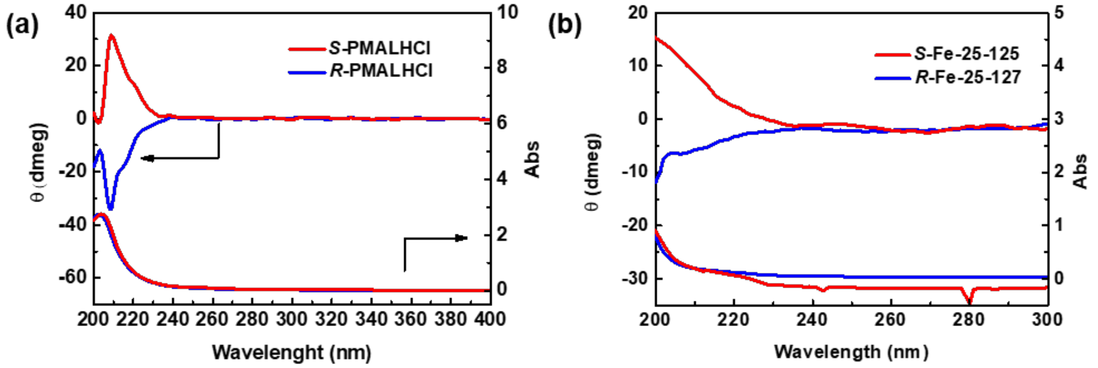

Supplementary Figure 4. CD spectrum and UV-vis spectrum of PMAL and nano-splitters. **a** CD spectrum (up) and UV-vis spectrum (down) of the solution of *R/S*-PMAL (DP=25), C (*R/S*-PMAL) = 0.01 mg mL^{-1}. **b** spectrum (up) and UV-vis spectrum (down) of *R/S*-nano-splitters (KBr plate).

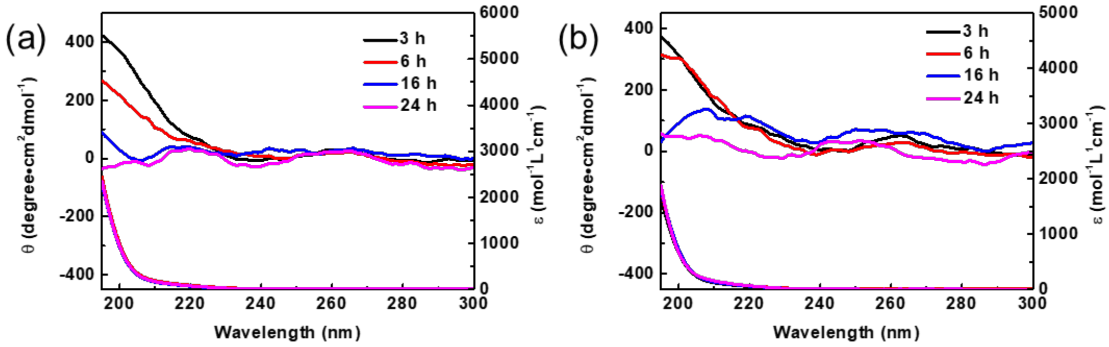

Supplementary Figure 5. CD spectra and UV-vis spectra of the supernatant over time. **a** CD spectrum (up) and UV-vis spectrum (down) of the supernatant when *S*-nano-splitter and *R*-seeds were added; **b** CD spectrum (up) and UV-vis spectrum (down) of the supernatant when only *R*-seeds were added.

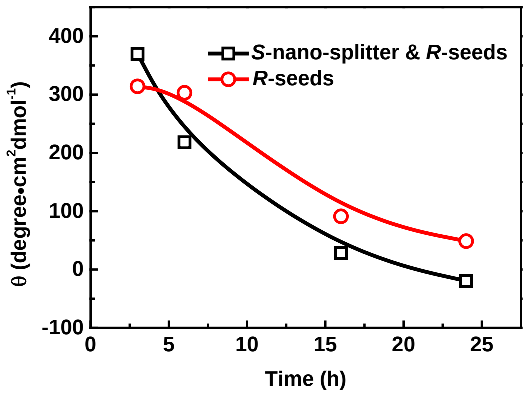

Supplementary Figure 6. CD signals at 200 nm over time.

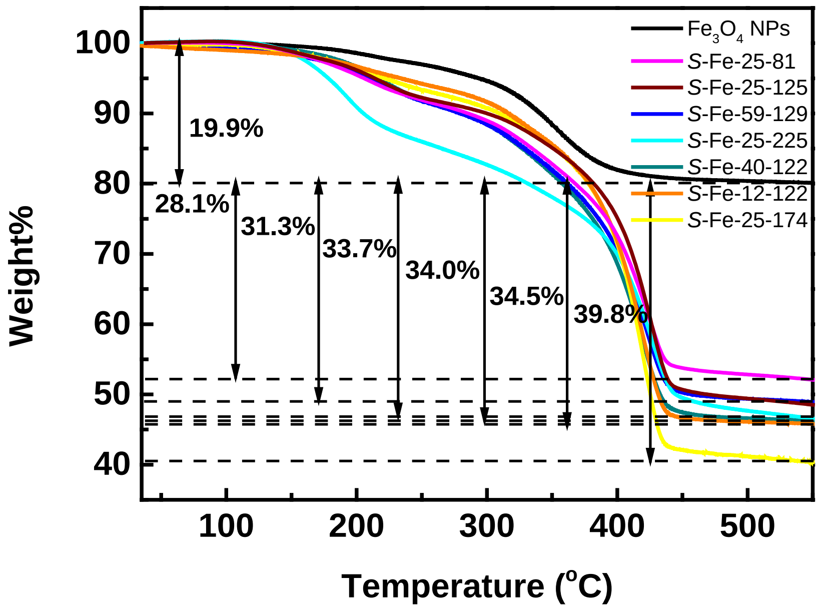

Supplementary Figure 7. TGA results of MNPs and *S*-Fe-m-n

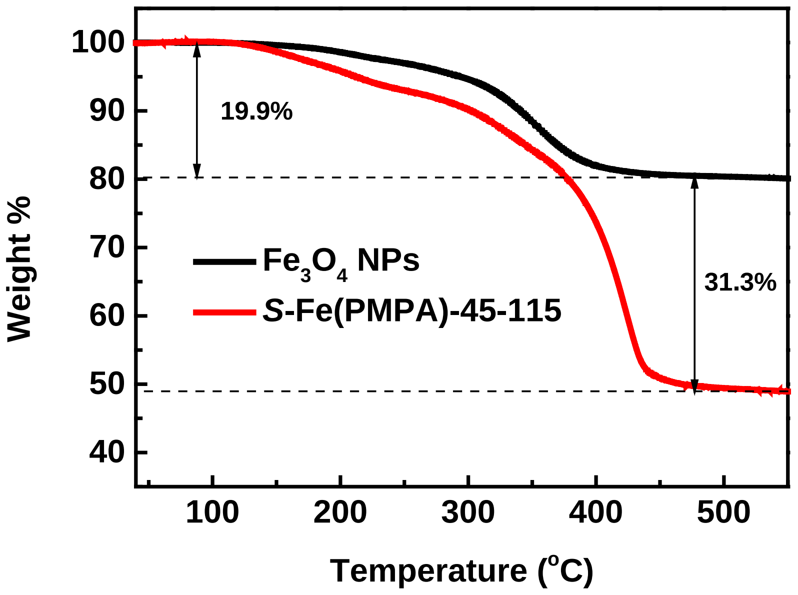

Supplementary Figure 8. TGA results of Fe_3O_4 NPs and *S*-Fe(PMAP)-45-115

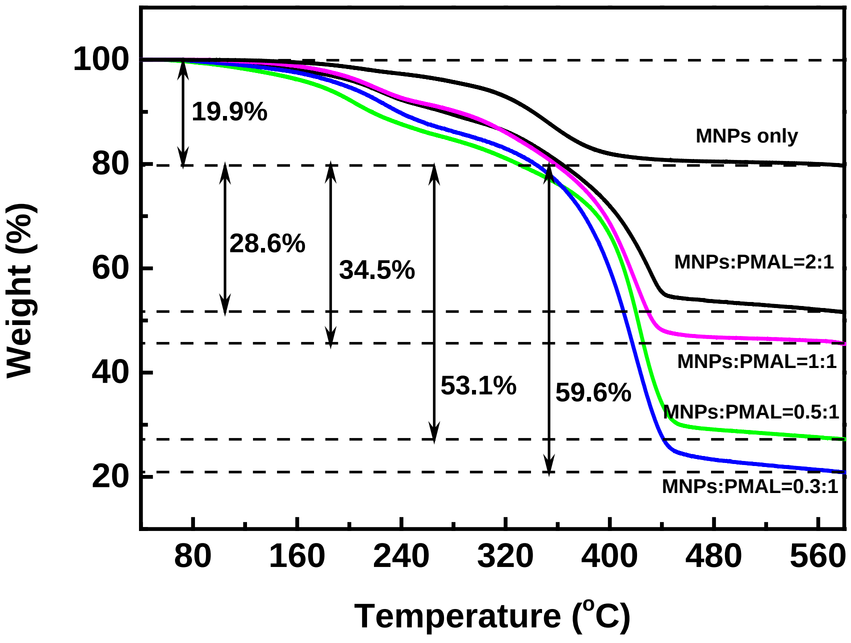

Supplementary Figure 9. TGA results of MNPs and *S*-Fe-40-122 with different composite ratio

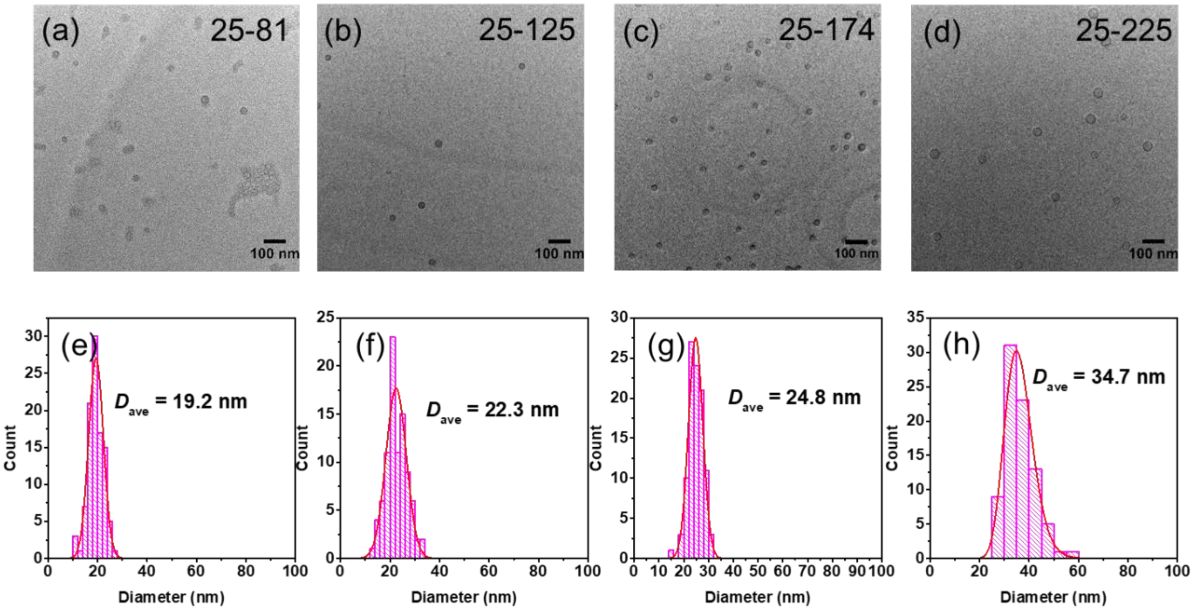

Supplementary Figure 10. The morphologies of the bare co-polymers in water. **a-d** The TEM images of the assemblies of PMAL$_m$-*b*-PSt$_n$ in water, where the m keeps constant and n changes. **e-h** The corresponding histograms of the diameter of each micelle, averaged over 100 micelles.

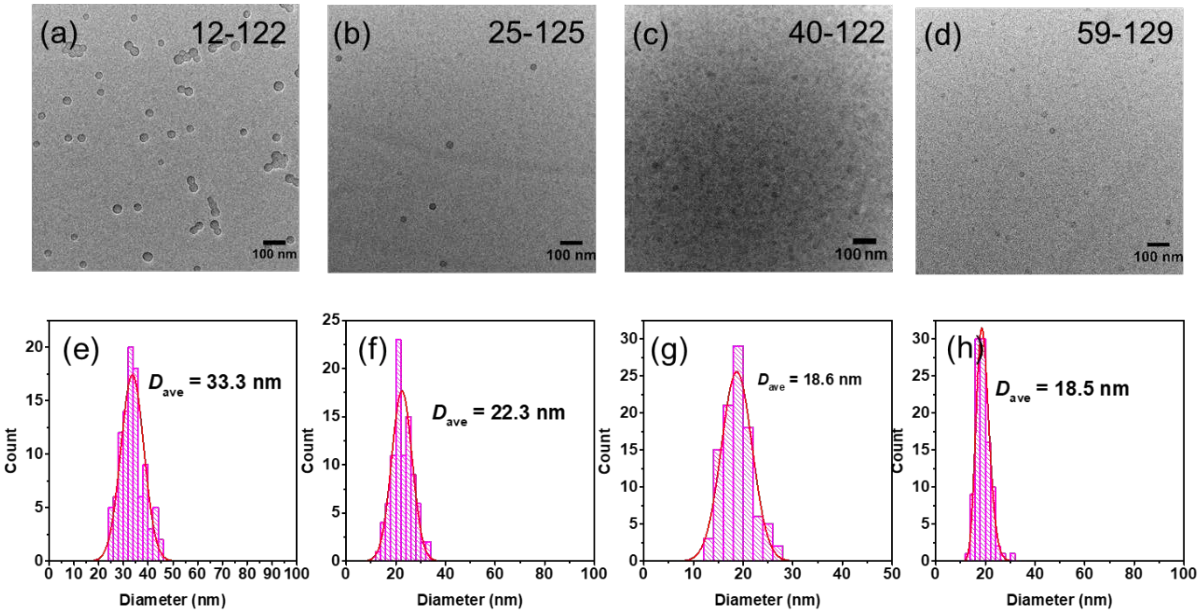

Supplementary Figure 11. The morphologies of the bare co-polymers in water. **a-d** The TEM images of the assemblies of PMAL$_m$-*b*-PSt$_n$ in water, where the n keeps close and m changes. **e-h** The corresponding histograms of the diameter of each micelle, averaged over 100 micelles.

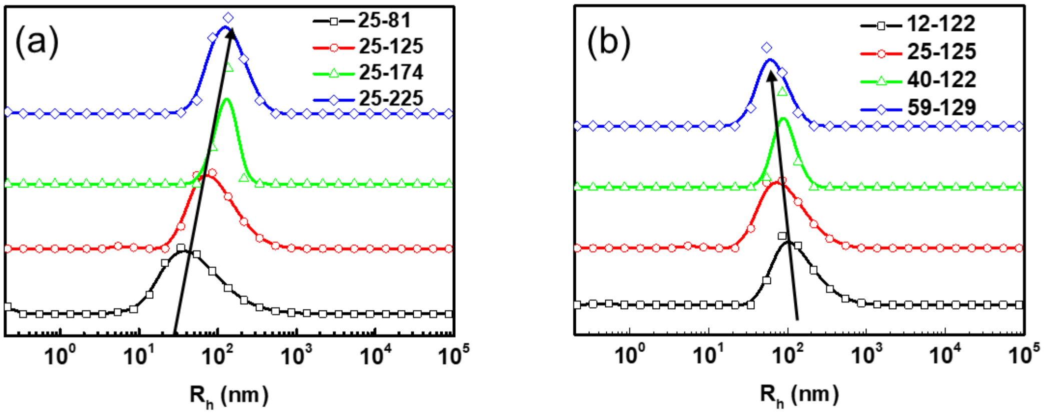

Supplementary Figure 12. The DLS results of the assemblies of $PMAL_m$-*b*-PSt_n at 90° in water. **a** The size distribution of the assemblies of $PMAL_m$-*b*-PSt_n, where the m keeps constant and m changes. **b** The size distribution of the assemblies of $PMAL_m$-*b*-PSt_n, where the n keeps close and m changes.

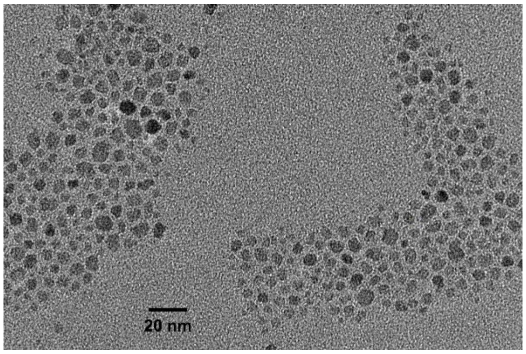

Supplementary Figure 13. The morphologies of the bare Fe_3O_4 MNPs

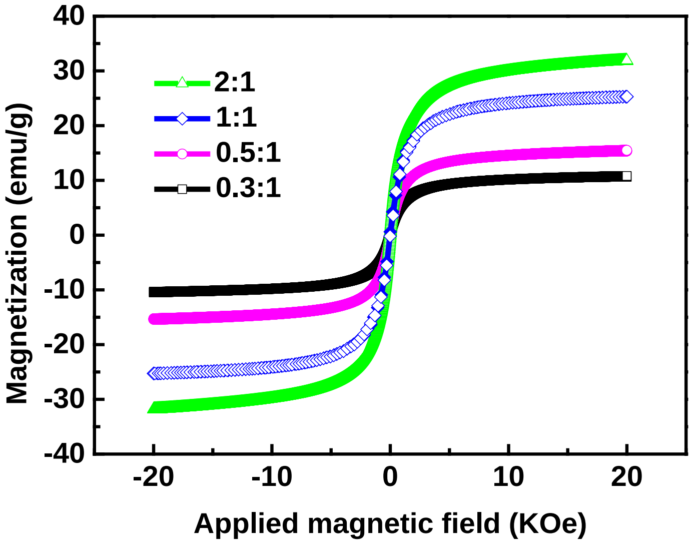

Supplementary Figure 14. The magnetic hysteresis loops of *S*-Fe-25-174 with different feeding ratio

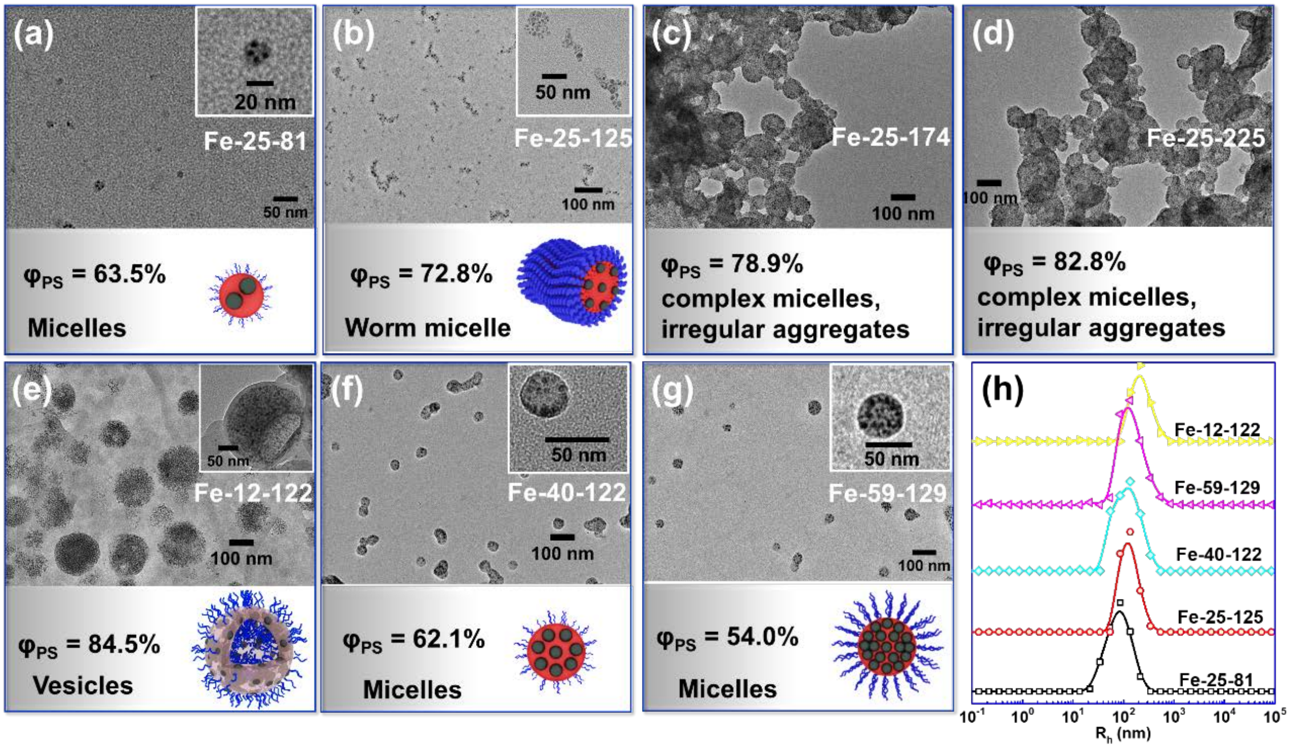

Supplementary Figure 15. The morphologies of the assemblies of nano-splitters. **a-g** Large-area of TEM images of the assemblies of different nano-splitters, and the 3D model of these assemblies (insert pictures: magnified images). **h** The DLS results of different nano-splitters at 90°.

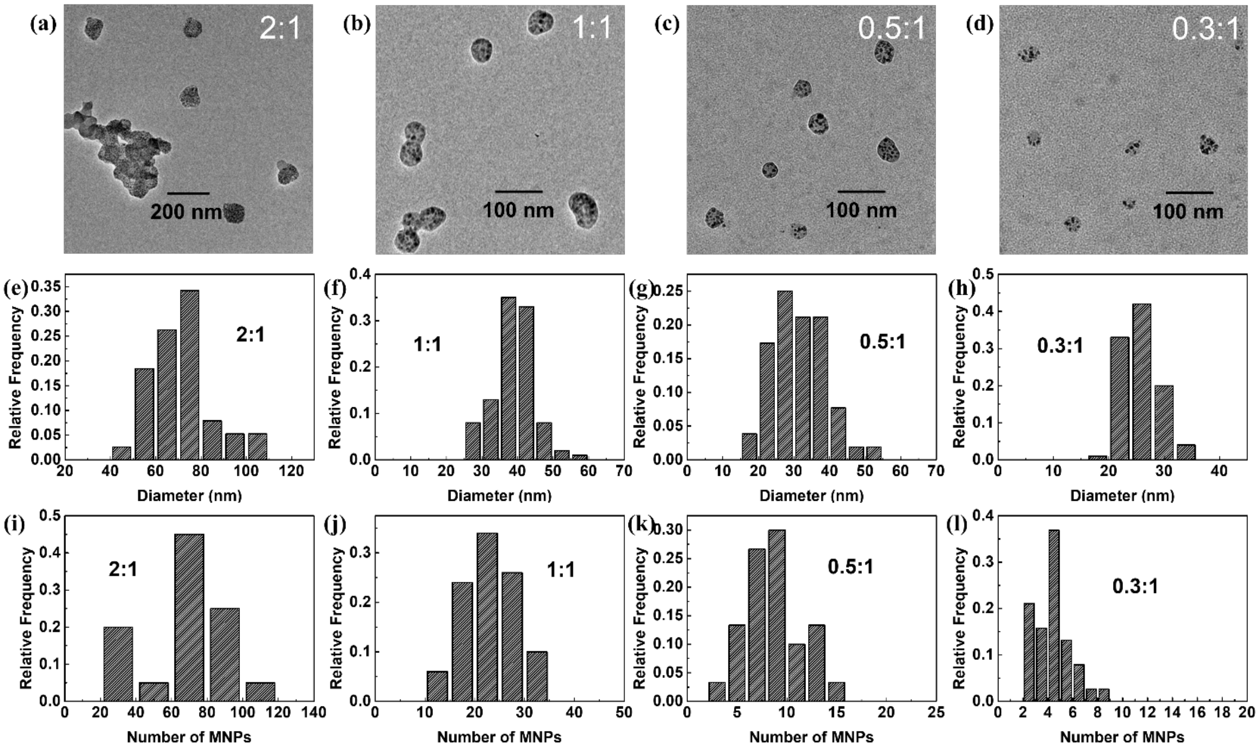

Supplementary Figure 16. The particle sizes of *S*-Fe-40-122. **a-d** TEM images of the assemblies of these nano-splitters (the ratio of NMPs : polymer is: a, 2:1; b, 1:1; c, 0.5:1; d, 0.3:1). **e-h** The corresponding histograms of the diameter of each micelle, averaged over 100 magnetomicelles (the ratio of NMPs : polymer is: e, 2:1; f, 1:1; g, 0.5:1; h, 0.3:1). **i-l** The corresponding histograms of the number of counted particles encapsulated within each micelle, averaged over 50 magnetomicelles (the ratio of NMPs : polymer is: i, 2:1; j, 1:1; k, 0.5:1; l, 0.3:1).

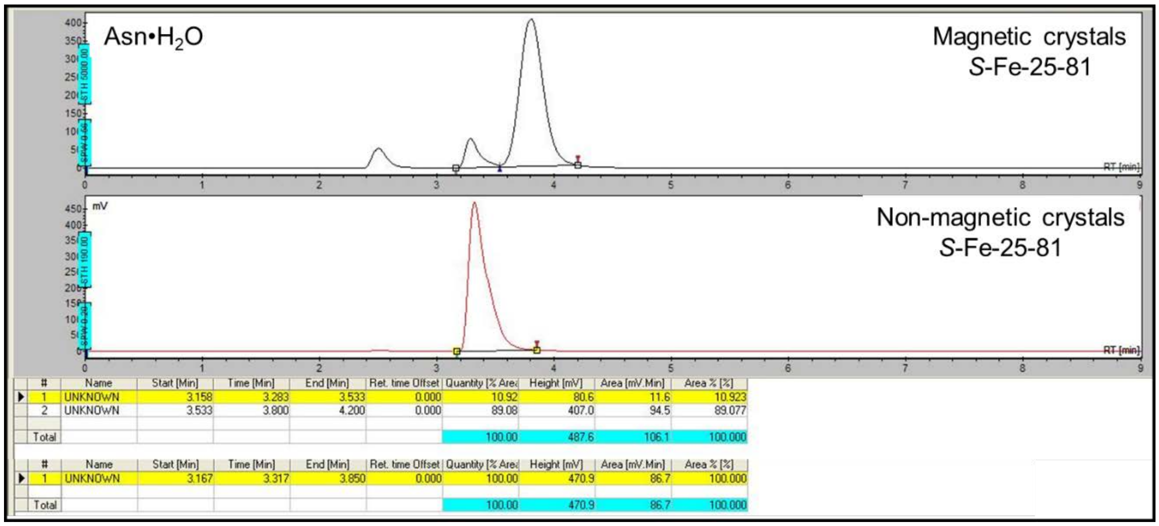

#	Name	Start [Min]	Time [Min]	End [Min]	Ret. time Offset	Quantity [% Area]	Height [mV]	Area [mV.Min]	Area % [%]
1	UNKNOWN	3.158	3.283	3.533	0.000	10.92	80.6	11.6	10.923
2	UNKNOWN	3.533	3.800	4.200	0.000	89.08	407.0	94.5	89.077
Total						100.00	487.6	106.1	100.000

#	Name	Start [Min]	Time [Min]	End [Min]	Ret. time Offset	Quantity [% Area]	Height [mV]	Area [mV.Min]	Area % [%]
1	UNKNOWN	3.167	3.317	3.850	0.000	100.00	470.9	86.7	100.000
Total						100.00	470.9	86.7	100.000

Supplementary Figure 17. Typical chiral HPLC results of the crystal Asn•H_2O mediated by *S*-Fe-25-81 at a weight concentration of 0.25 wt%

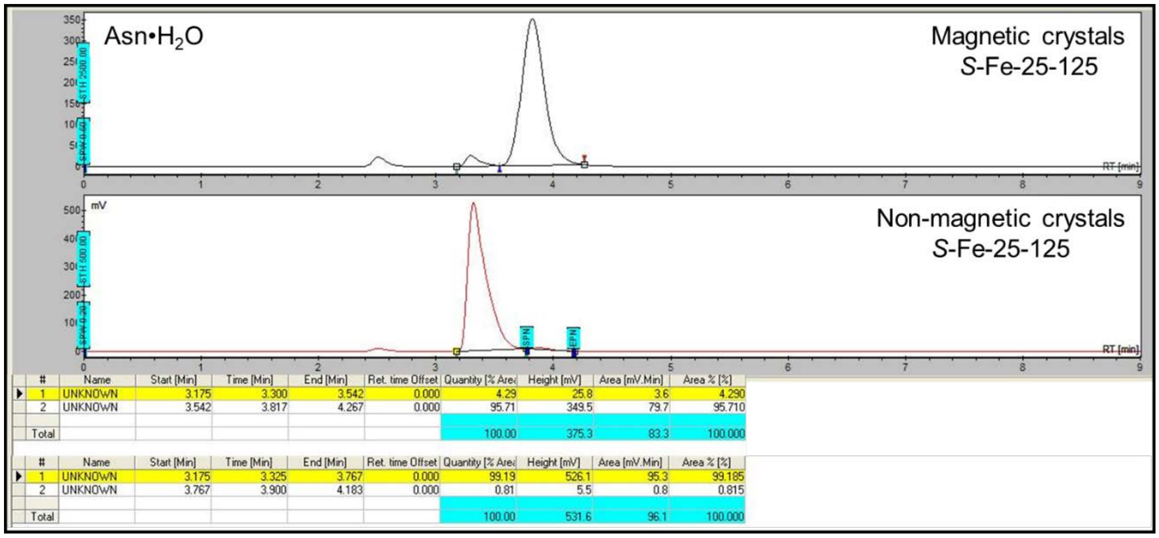

#	Name	Start [Min]	Time [Min]	End [Min]	Ret. time Offset	Quantity [% Area]	Height [mV]	Area [mV.Min]	Area % [%]
1	UNKNOWN	3.175	3.300	3.542	0.000	4.29	25.8	3.6	4.290
2	UNKNOWN	3.542	3.817	4.267	0.000	95.71	349.5	79.7	95.710
Total						100.00	375.3	83.3	100.000

#	Name	Start [Min]	Time [Min]	End [Min]	Ret. time Offset	Quantity [% Area]	Height [mV]	Area [mV.Min]	Area % [%]
1	UNKNOWN	3.175	3.325	3.767	0.000	99.19	526.1	95.3	99.185
2	UNKNOWN	3.767	3.900	4.183	0.000	0.81	5.5	0.8	0.815
Total						100.00	531.6	96.1	100.000

Supplementary Figure 18. Typical chiral HPLC results of the crystal Asn•H_2O mediated by *S*-Fe-25-125 at 0.25 wt%

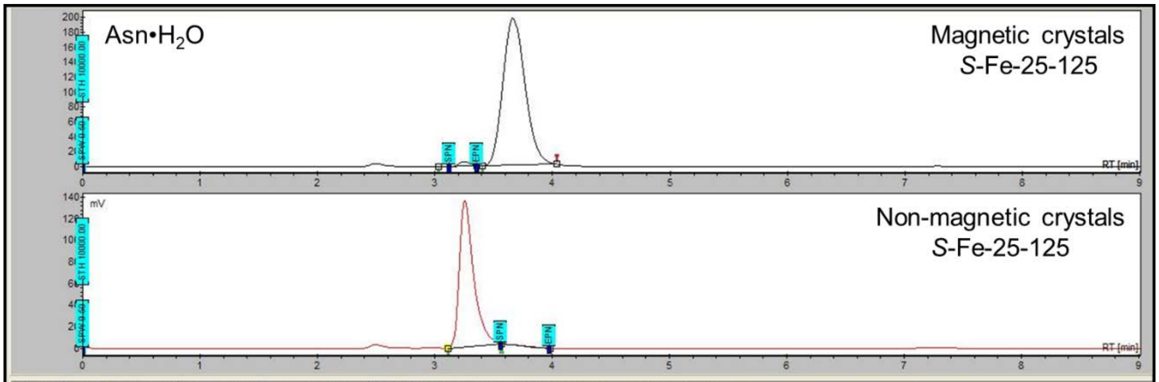

#	Name	Start [Min]	Time [Min]	End [Min]	Ret. time Offset	Quantity [% Area]	Height [mV]	Area [mV.Min]	Area % [%]
1	UNKNOWN	3.033	3.250	3.358	0.000	1.25	5.8	0.5	1.246
2	UNKNOWN	3.400	3.667	4.033	0.000	98.75	197.5	42.9	98.754
Total						100.00	203.3	43.5	100.000

#	Name	Start [Min]	Time [Min]	End [Min]	Ret. time Offset	Quantity [% Area]	Height [mV]	Area [mV.Min]	Area % [%]
1	UNKNOWN	3.117	3.258	3.567	0.000	99.35	135.1	19.4	99.354
2	UNKNOWN	3.567	3.833	3.983	0.000	0.65	0.7	0.1	0.646
Total						100.00	135.9	19.6	100.000

Supplementary Figure 19. Chiral HPLC results of the crystal Asn•H_2O mediated by *S*-Fe-25-125 at a weight concentration of 0.5 wt% and the solvent was slowly evaporated after the dark brown crystals appeared.

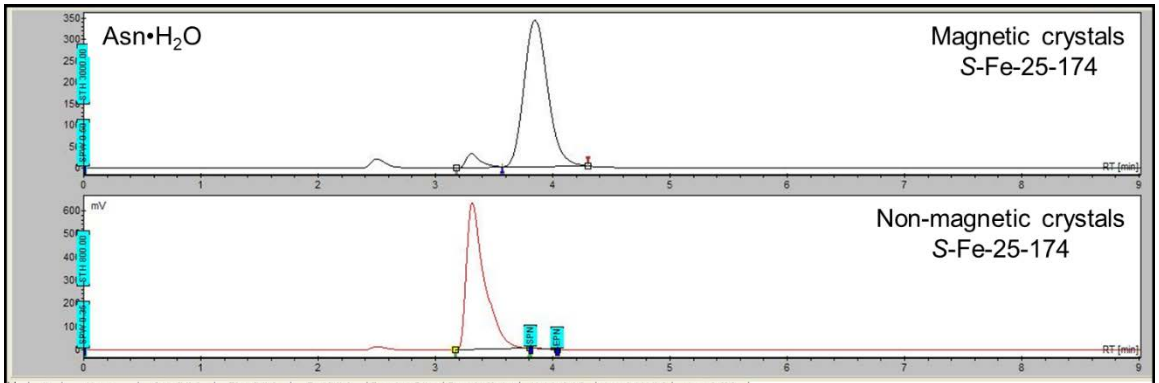

#	Name	Start [Min]	Time [Min]	End [Min]	Ret. time Offset	Quantity [% Area]	Height [mV]	Area [mV.Min]	Area % [%]
1	UNKNOWN	3.183	3.308	3.567	0.000	5.59	32.7	4.7	5.587
2	UNKNOWN	3.567	3.850	4.300	0.000	94.41	342.1	79.6	94.413
Total						100.00	374.8	84.3	100.000

#	Name	Start [Min]	Time [Min]	End [Min]	Ret. time Offset	Quantity [% Area]	Height [mV]	Area [mV.Min]	Area % [%]
1	UNKNOWN	3.167	3.317	3.800	0.000	99.93	631.9	113.9	99.927
2	UNKNOWN	3.800	3.883	4.042	0.000	0.07	0.9	0.1	0.073
Total						100.00	632.8	114.0	100.000

Supplementary Figure 20. Typical chiral HPLC results of the crystal Asn•H_2O mediated by *S*-Fe-25-174 at 0.25 wt%

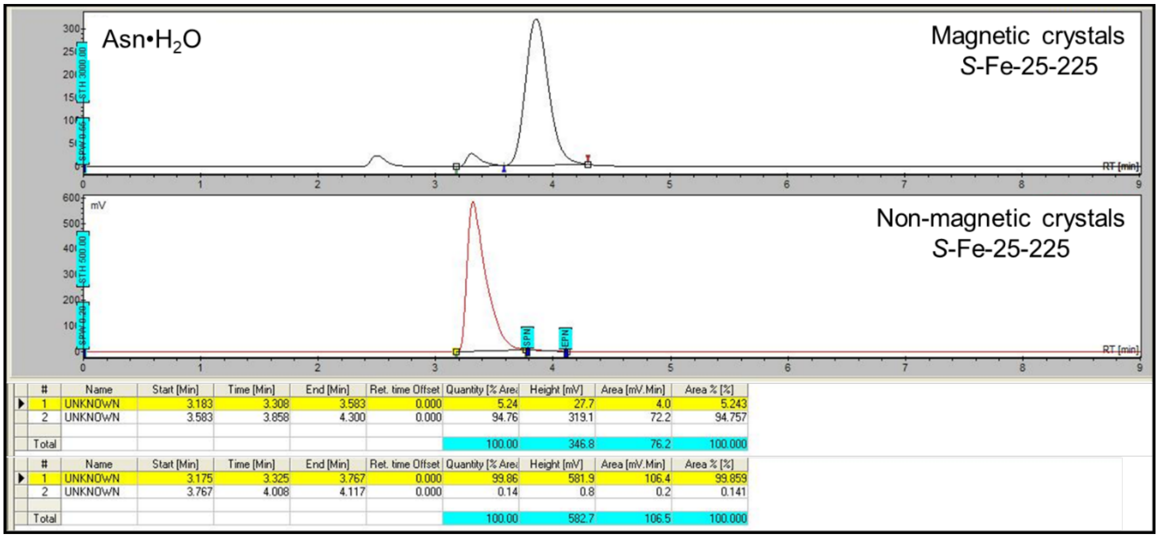

Supplementary Figure 21. Typical chiral HPLC results of the crystal Asn•H_2O mediated by *S*-Fe-25-225 at 0.25 wt%

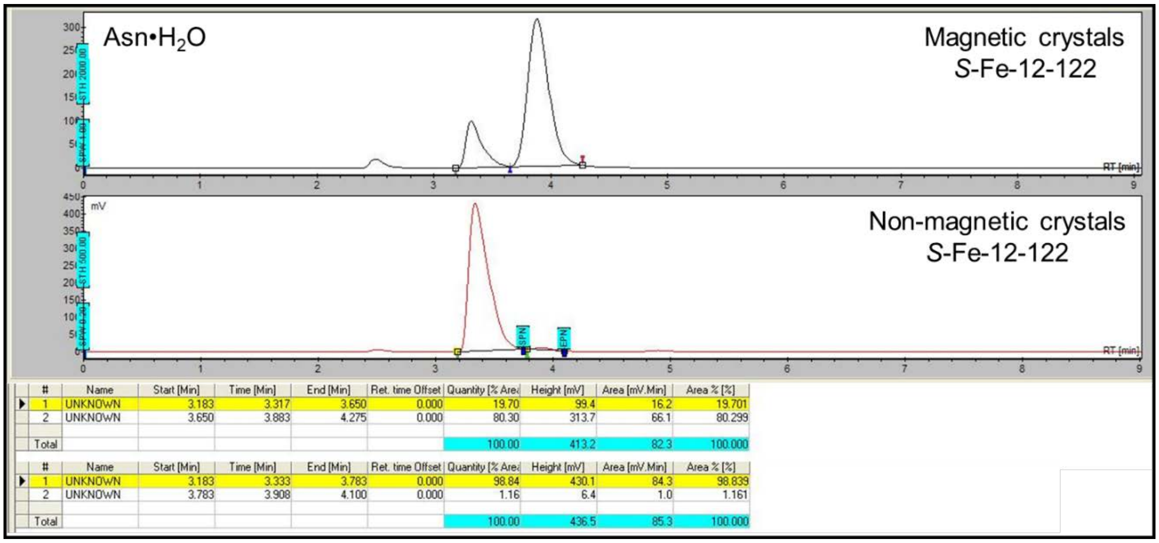

Supplementary Figure 22. Typical chiral HPLC results of the crystal Asn•H_2O mediated by *S*-Fe-12-122 at 0.25 wt%

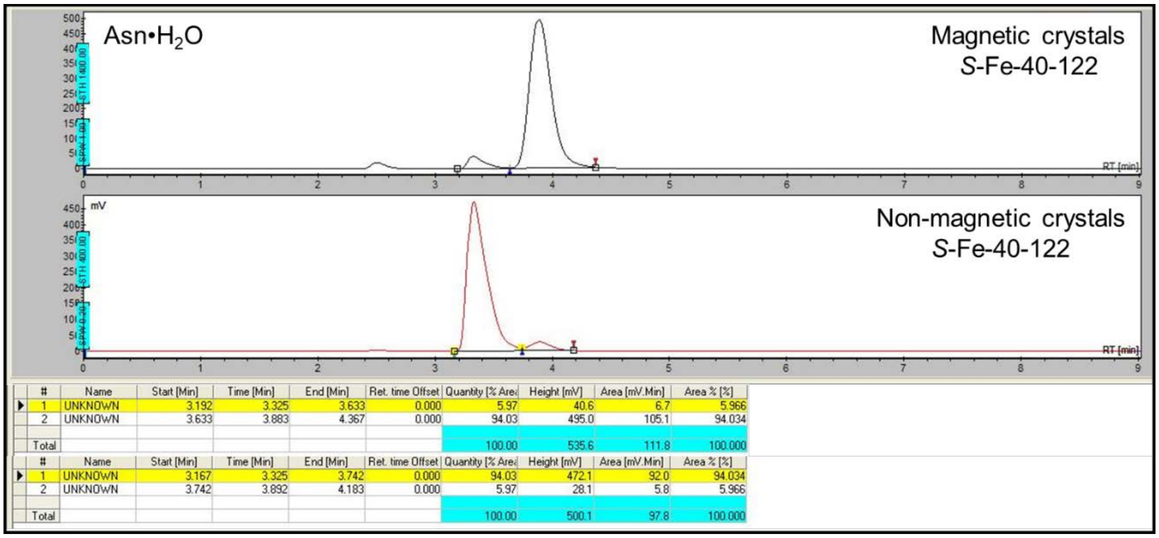

#	Name	Start [Min]	Time [Min]	End [Min]	Ret. time Offset	Quantity [% Are	Height [mV]	Area [mV.Min]	Area % [%]
1	UNKNOWN	3.192	3.325	3.633	0.000	5.97	40.6	6.7	5.966
2	UNKNOWN	3.633	3.883	4.367	0.000	94.03	495.0	105.1	94.034
Total						100.00	535.6	111.8	100.000

#	Name	Start [Min]	Time [Min]	End [Min]	Ret. time Offset	Quantity [% Are	Height [mV]	Area [mV.Min]	Area % [%]
1	UNKNOWN	3.167	3.325	3.742	0.000	94.03	472.1	92.0	94.034
2	UNKNOWN	3.742	3.892	4.183	0.000	5.97	28.1	5.8	5.966
Total						100.00	500.1	97.8	100.000

Supplementary Figure 23. Typical chiral HPLC results of the crystal Asn•H_2O mediated by *S*-Fe-40-122 at 0.25 wt%

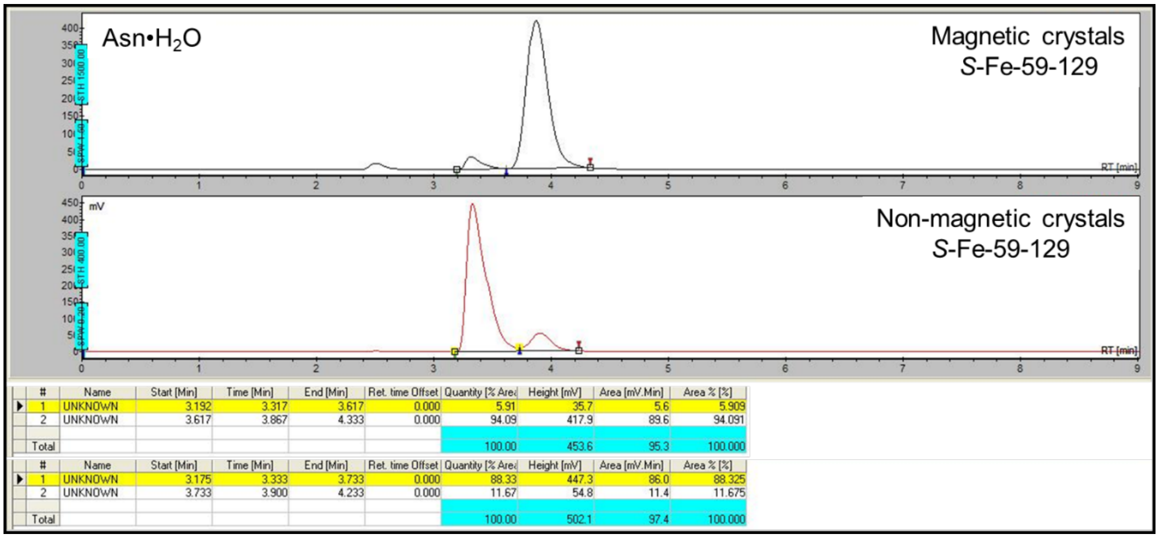

#	Name	Start [Min]	Time [Min]	End [Min]	Ret. time Offset	Quantity [% Are	Height [mV]	Area [mV.Min]	Area % [%]
1	UNKNOWN	3.192	3.317	3.617	0.000	5.91	35.7	5.6	5.909
2	UNKNOWN	3.617	3.867	4.333	0.000	94.09	417.9	89.6	94.091
Total						100.00	453.6	95.3	100.000

#	Name	Start [Min]	Time [Min]	End [Min]	Ret. time Offset	Quantity [% Are	Height [mV]	Area [mV.Min]	Area % [%]
1	UNKNOWN	3.175	3.333	3.733	0.000	88.33	447.3	86.0	88.325
2	UNKNOWN	3.733	3.900	4.233	0.000	11.67	54.8	11.4	11.675
Total						100.00	502.1	97.4	100.000

Supplementary Figure 24. Typical chiral HPLC results of the crystal Asn•H_2O mediated by *S*-Fe-59-129 at 0.25 wt%.

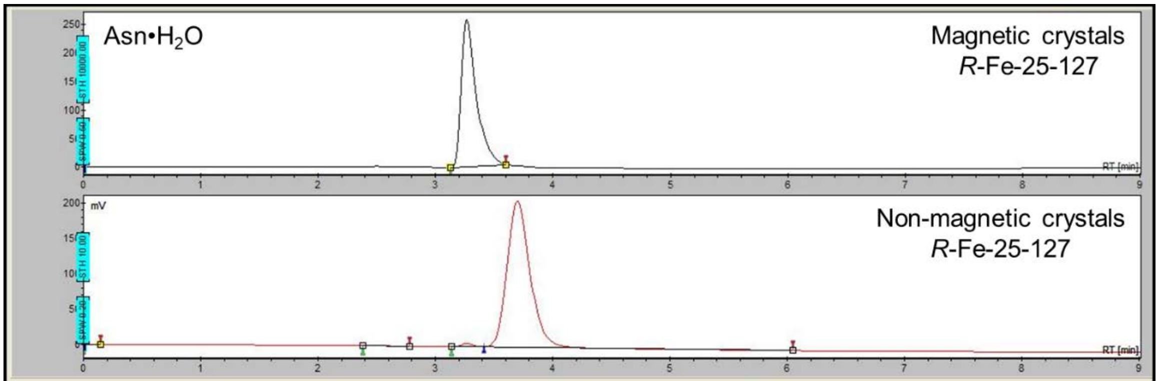

#	Name	Start [Min]	Time [Min]	End [Min]	Ret. time Offset	Quantity [% Area]	Height [mV]	Area [mV.Min]	Area % [%]
1	UNKNOWN	3.125	3.267	3.600	0.000	100.00	257.6	38.7	100.000
Total						100.00	257.6	38.7	100.000

#	Name	Start [Min]	Time [Min]	End [Min]	Ret. time Offset	Quantity [% Area]	Height [mV]	Area [mV.Min]	Area % [%]
1	UNKNOWN	3.067	3.258	3.383	0.000	0.96	4.5	0.4	0.958
2	UNKNOWN	3.433	3.700	4.067	0.000	99.04	204.8	45.9	99.042
Total						100.00	209.4	46.3	100.000

Supplementary Figure 25. Typical chiral HPLC results of the crystal Asn•H_2O mediated by *R*-Fe-25-127 at 0.25 wt%.

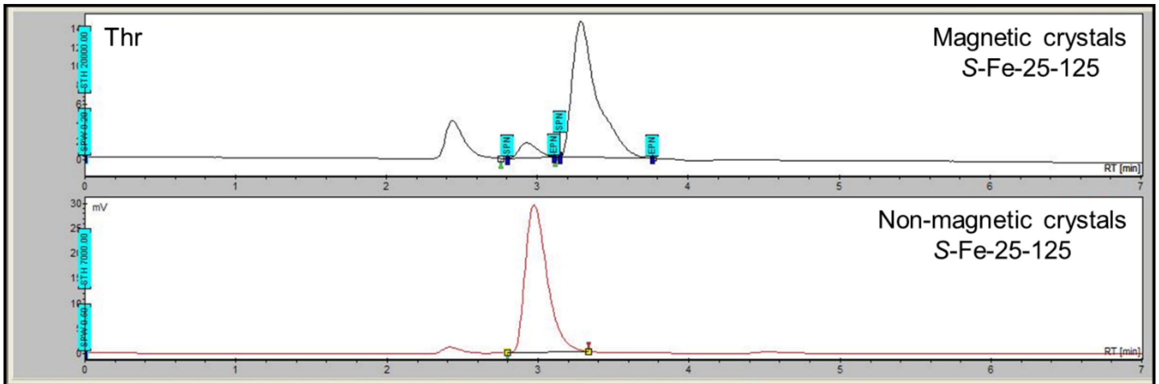

#	Name	Start [Min]	Time [Min]	End [Min]	Ret. time Offset	Quantity [% Area]	Height [mV]	Area [mV.Min]	Area % [%]
1	UNKNOWN	2.758	2.925	3.117	0.000	7.36	1.6	0.2	7.361
2	UNKNOWN	3.117	3.292	3.767	0.000	92.64	14.6	2.8	92.639
Total						100.00	16.2	3.0	100.000

#	Name	Start [Min]	Time [Min]	End [Min]	Ret. time Offset	Quantity [% Area]	Height [mV]	Area [mV.Min]	Area % [%]
1	UNKNOWN	2.800	2.975	3.333	0.000	100.00	29.5	4.9	100.000
Total						100.00	29.5	4.9	100.000

Supplementary Figure 26. Typical chiral HPLC results of the crystal Thr mediated by *S*-Fe-25-125 at 0.25 wt%.

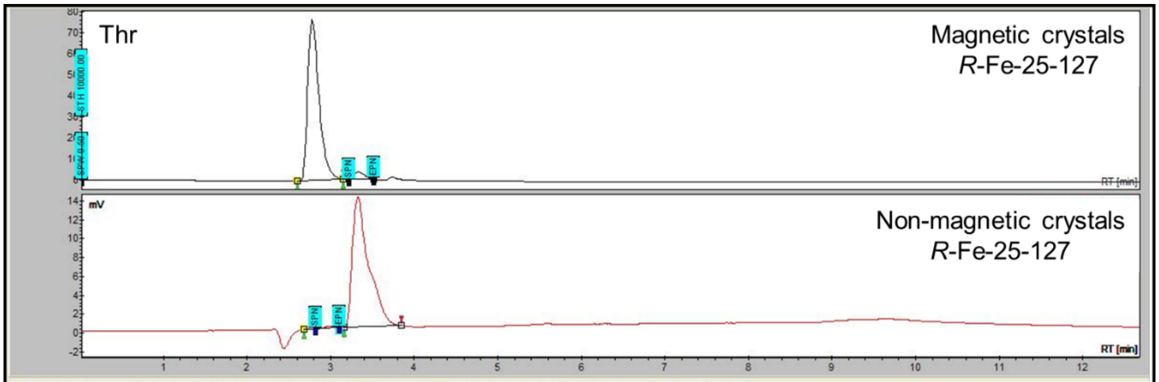

#	Name	Start [Min]	Time [Min]	End [Min]	Ret. time Offset	Quantity [% Area]	Height [mV]	Area [mV.Min]	Area % [%]
1	UNKNOWN	2.283	2.442	2.767	0.000	95.91	76.4	10.6	95.912
2	UNKNOWN	2.767	2.933	3.100	0.000	4.09	3.6	0.5	4.088
Total						100.00	79.9	11.1	100.000

#	Name	Start [Min]	Time [Min]	End [Min]	Ret. time Offset	Quantity [% Area]	Height [mV]	Area [mV.Min]	Area % [%]
1	UNKNOWN	2.683	2.975	3.108	0.000	0.73	0.2	0.0	0.730
2	UNKNOWN	3.158	3.325	3.850	0.000	99.27	13.8	3.1	99.270
Total						100.00	13.9	3.2	100.000

Supplementary Figure 27. Typical chiral HPLC results of the crystal Thr mediated by *R*-Fe-25-127 at 0.25 wt%.

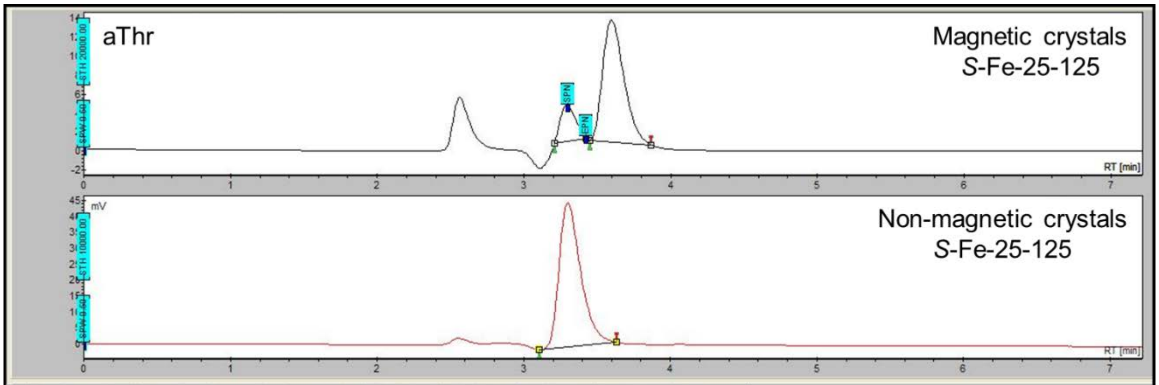

#	Name	Start [Min]	Time [Min]	End [Min]	Ret. time Offset	Quantity [% Area]	Height [mV]	Area [mV.Min]	Area % [%]
1	UNKNOWN	3.208	3.292	3.425	0.000	17.52	3.8	0.5	17.524
2	UNKNOWN	3.450	3.600	3.867	0.000	82.48	12.9	2.2	82.476
Total						100.00	16.6	2.6	100.000

#	Name	Start [Min]	Time [Min]	End [Min]	Ret. time Offset	Quantity [% Area]	Height [mV]	Area [mV.Min]	Area % [%]
1	UNKNOWN	3.108	3.300	3.633	0.000	100.00	45.4	7.8	100.000
Total						100.00	45.4	7.8	100.000

Supplementary Figure 28. Typical chiral HPLC results of the crystal aThr mediated by *S*-Fe-25-125 at 0.25 wt%.

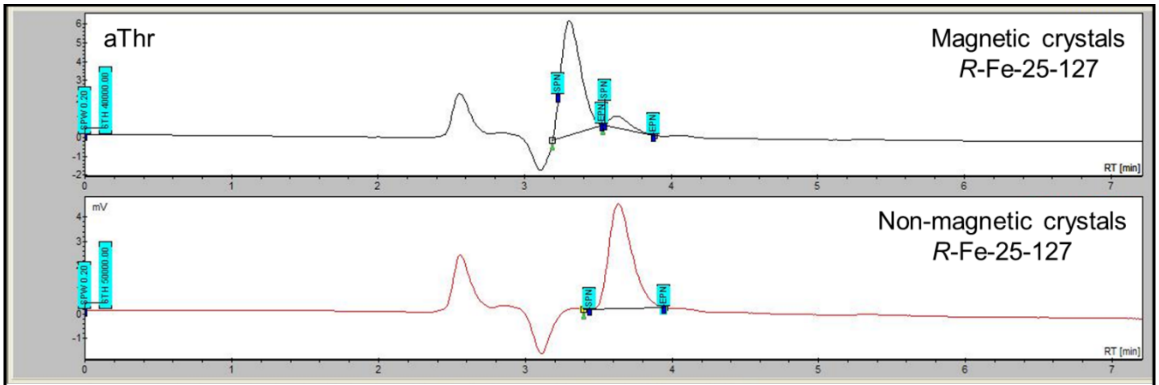

#	Name	Start [Min]	Time [Min]	End [Min]	Ret. time Offset	Quantity [% Area]	Height [mV]	Area [mV.Min]	Area % [%]
1	UNKNOWN	3.192	3.300	3.533	0.000	90.94	6.1	0.9	90.938
2	UNKNOWN	3.533	3.633	3.883	0.000	9.06	0.6	0.1	9.062
Total						100.00	6.7	1.0	100.000

#	Name	Start [Min]	Time [Min]	End [Min]	Ret. time Offset	Quantity [% Area]	Height [mV]	Area [mV.Min]	Area % [%]
1	UNKNOWN	3.400	3.633	3.950	0.000	100.00	4.3	0.8	100.000
Total						100.00	4.3	0.8	100.000

Supplementary Figure 29. Typical chiral HPLC results of the crystal aThr mediated by *R*-Fe-25-127 at 0.25 wt%

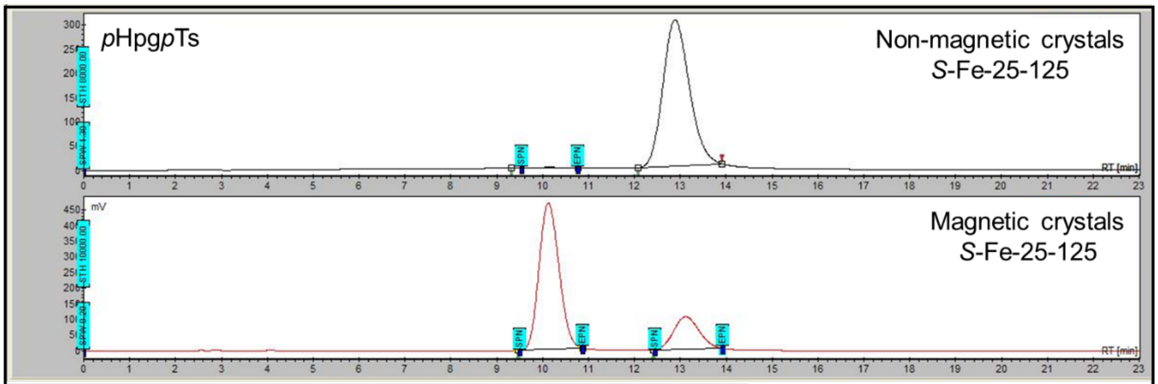

#	Name	Start [Min]	Time [Min]	End [Min]	Ret. time Offset	Quantity [% Area]	Height [mV]	Area [mV.Min]	Area % [%]
1	UNKNOWN	9.317	10.150	10.775	0.000	0.42	1.8	0.9	0.420
2	UNKNOWN	12.083	12.883	13.917	0.000	99.58	301.6	201.9	99.580
Total						100.00	303.4	202.8	100.000

#	Name	Start [Min]	Time [Min]	End [Min]	Ret. time Offset	Quantity [% Area]	Height [mV]	Area [mV.Min]	Area % [%]
1	UNKNOWN	9.467	10.125	10.875	0.000	77.79	468.3	236.3	77.791
2	UNKNOWN	12.408	13.117	13.933	0.000	22.21	104.0	67.5	22.209
Total						100.00	572.3	303.7	100.000

Supplementary Figure 30. Typical chiral HPLC results of the crystal *p*Hpg*p*Ts mediated by *S*-Fe(PMPA)-45-115 at 0.25 wt%

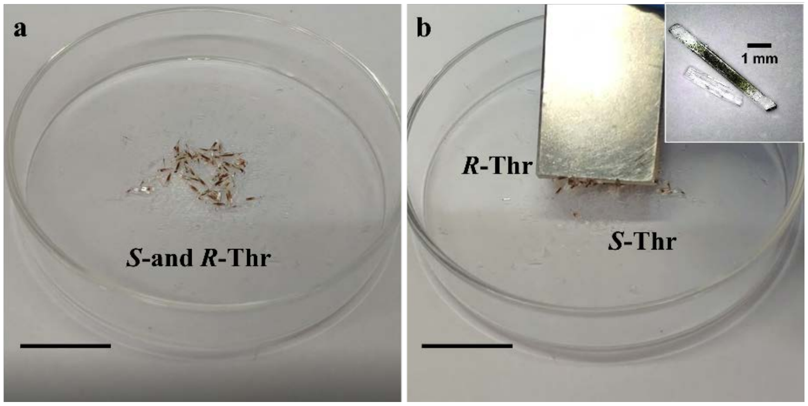

Supplementary Figure 31. Typical images of Thr crystal mediated by *S*-Fe-25-125. **a** The mixture crystals of *R* and *S*-Thr. **b** The separated crystals by applying a magnetic field. Scale bars: 2 cm (**a-b**).

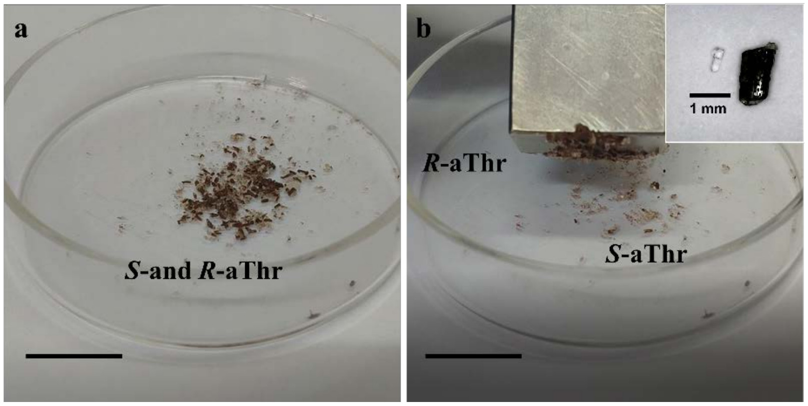

Supplementary Figure 32. Typical images of aThr crystal mediated by *S*-Fe-25-125. **a** The mixture crystals of *R* and *S*-aThr. **b** The separated crystals by applying a magnetic field. Scale bars: 2 cm (**a-b**).

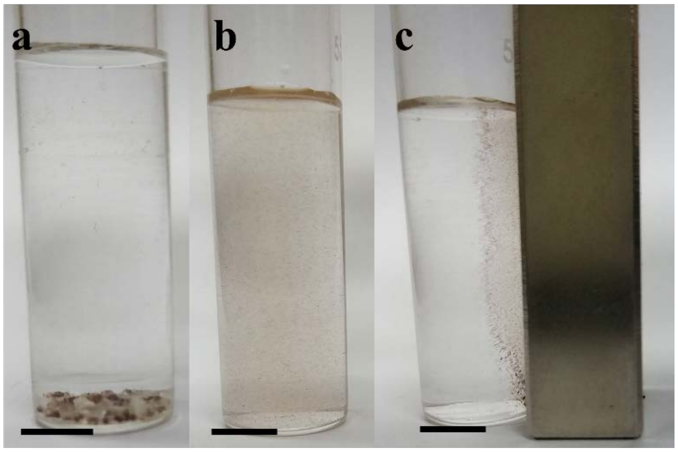

Supplementary Figure 33. Recycling of nano-splitters in crystals. **a** Magnetic crystals in water. **b** Magnetic crystals dissolve in water. **c** Separability of the nano-splitters by placing an external magnetic field. Scale bars: 1 cm (**a-c**).

Supplementary Table 1. RAFT polymerization results of CDP-*S*-PMALBoc[a], CDP-*R*-PMALBoc (P5), and CDP-*S*-PMPABoc (P6)[b]

	Feeding ratio			Time (h)	Yield (%)	$M_{n,\,GPC}$[c]	PDI
	M	CDP	AIBN				
P1	200	10	1	12	53	3.7 k	1.12
P2	500	10	1	18	66	8.2 k	1.21
P3	800	10	1	16	72	12.6 k	1.24
P4	1000	10	1	18	87	18.6 k	1.24
P5	500	10	1	18	73	8.5 k	1.19
P6	500	5	1	48	55	15.7 k	1.28

[a] Monomer concentration = 0.16 g mL^{-1}; temperature, 65 ^{o}C; solvent, dioxane. [b] Monomer concentration = 0.08 g mL^{-1}; temperature, 80 ^{o}C; solvent, dioxane. [c] Obtained from the corresponding $PMALBocOCH_3$ and $PMPABocOCH_3$.

Supplementary Table 2. The co-polymerization results of *S*-$PMALBoc_m$-*b*-PSt_n, *R*-$PMALBoc_m$-*b*-PSt_n (C8), and *S*-$PMPABoc_m$-*b*-PSt_n (C9)[a]

Run	Time (h)	Mn ($*10^4$)[b]	PDI	m	n
C1	3	1.70	1.30	25	81
C2	8	2.11	1.24	25	125
C3	10	2.66	1.29	25	174
C4	12	3.16	1.14	25	225
C5	9	1.65	1.17	12	122
C6	7.5	2.53	1.26	40	122
C7	9.5	3.20	1.26	59	129
C8	9	2.19	1.21	25	127
C9	12	2.77	1.40	45	115

[a] St concentration = 0.16 g mL^{-1}; [St]:[Macro-CDP]:[AIBN]=10000:10:1; temperature, 80 ^{o}C; solvent, dioxane. [b] Obtained from the corresponding $PMALBocOCH_3$-*b*-PSt and $PMPABocOCH_3$-*b*-PSt.

Supplementary Table 3. Comparison of the properties of the assemblies of $PMAL_m$-*b*-PSt_n and Fe-m-n

	Polymers only			Polymers & Fe_3O_4 MNPs				
	φ_{PSt} (%)	Shape	D_{ave} (nm)	Shape	φ_{NP} (%)	D_{ave} (nm)	N_{NPs}(obs)	N_{NPs}(cal)
**$PMAL_{25}$-*b*-PSt_{81}**	63.5	Sphere	23.7	Micelles	16.4	20.0	2	6
**$PMAL_{25}$-*b*-PSt_{125}**	72.8	Sphere	13.4	Worm-like micelles	14.8	-	-	-
**$PMAL_{25}$-*b*-PSt_{174}**	78.9	Sphere	16.5	complex micelles and Vesicles	11.9	-	-	-
**$PMAL_{25}$-*b*-PSt_{225}**	82.8	Sphere	25.4	Irregular aggregates	14.6	-	-	-
**$PMAL_{12}$-*b*-PSt_{122}**	84.5	Sphere	10.9	Vesicles	12.8	-	-	-
**$PMAL_{40}$-*b*-PSt_{122}**	62.1	Sphere	18.6	2:1 Micelles	17.6	71.5	69	297
				1:1 Micelles	14.8	38.4	22	38
				0.5:1 Micelles	7.2	31.3	8	11
				0.3:1 Micelles	5.1	25.8	4	4
**$PMAL_{59}$-*b*-PSt_{129}**	54.0	sphere	20.3	Micelles	16.3	45.9	30	72

Supplementary References:

1 Li, W. K. *et al.* A simple route to improve inorganic nanoparticles loading efficiency in block copolymer micelles. *Macromolecules* **46**, 2282-2291 (2013).
