# Supplementary material for: Enantiomer-selective magnetization of conglomerates for quantitative chiral separation
Source: Nat Commun. 2019 Apr 29;10:1964. doi: 10.1038/s41467-019-09997-y (PMC6488659; doi:10.1038/s41467-019-09997-y)
Supplement: Supplementary file 3 — Description of Additional Supplementary Files [file 41467_2019_9997_MOESM3_ESM.pdf]

Description of Additional Supplementary Files

File name: Supplementary Movie 1

Description: The magnetic separation process of *rac*-Asn•H_2O by using *S*-Fe-25-125 as additive.

File name: Supplementary Movie 2

Description: The magnetic separation process of *rac*-Asn•H_2O by using *R*-Fe-25-127 as additive.
